# Novel Anti-HER2 Antibody-Drug Conjugates Versus T-DM1 for HER2-Positive Metastatic Breast Cancer After Tyrosine Kinase Inhibitors Treatment

**DOI:** 10.1093/oncolo/oyad127

**Published:** 2023-05-22

**Authors:** Chenchen Ji, Feng Li, Yang Yuan, Huiqiang Zhang, Li Bian, Shaohua Zhang, Tao Wang, Jianbin Li, Zefei Jiang

**Affiliations:** Department of Breast Oncology, The Fifth Medical Center of Chinese PLA General Hospital, Beijing, People’s Republic of China; Department of Breast Oncology, The Fifth Medical Center of Chinese PLA General Hospital, Beijing, People’s Republic of China; Department of Breast Oncology, The Fifth Medical Center of Chinese PLA General Hospital, Beijing, People’s Republic of China; Department of Breast Oncology, The Fifth Medical Center of Chinese PLA General Hospital, Beijing, People’s Republic of China; Department of Breast Oncology, The Fifth Medical Center of Chinese PLA General Hospital, Beijing, People’s Republic of China; Department of Breast Oncology, The Fifth Medical Center of Chinese PLA General Hospital, Beijing, People’s Republic of China; Department of Breast Oncology, The Fifth Medical Center of Chinese PLA General Hospital, Beijing, People’s Republic of China; Department of Breast Oncology, The Fifth Medical Center of Chinese PLA General Hospital, Beijing, People’s Republic of China; Department of Medical Molecular Biology, Beijing Institute of Biotechnology, Academy of Military Medical Sciences, Beijing, People’s Republic of China; Department of Breast Oncology, The Fifth Medical Center of Chinese PLA General Hospital, Beijing, People’s Republic of China

**Keywords:** metastatic breast cancer, tyrosine kinase inhibitor, antibody-drug conjugates, trastuzumab emtansine, trastuzumab deruxtecan

## Abstract

**Background:**

Antibody-drug conjugates (ADCs) have been the preferred regimens for human epidermal growth factor receptor 2 (HER2)-positive metastatic breast cancer (MBC) after trastuzumab. Unfortunately, there is little data showing which ADCs should be chosen for those patients whose treatment with tyrosine kinase inhibitors (TKIs) failed. This study aims to analyze the efficacy and safety between novel anti-HER2 ADCs and trastuzumab emtansine (T-DM1) for those with TKIs failure.

**Materials and methods:**

HER2-positive MBC using ADCs from January 2013 to June 2022 were included, and all of them were treated with TKIs. The primary study endpoint was progression-free survival (PFS), and the secondary study endpoints were objective response rate (ORR), clinical benefit rate (CBR), and safety.

**Results:**

A total of 144 patients with 73 patients in the novel anti-HER2 ADCs group and 71 patients in the T-DM1 group. In these novel ADCs, 30 patients received trastuzumab deruxtecan (T-Dxd), 43 patients receive other novel ADCs. The median PFS in the novel ADCs group and T-DM1 group were 7.0 months versus 4.0 months, respectively, and ORR was 54.8% versus 22.5%, CBR was 65.8% versus 47.9%, respectively. In subgroups analysis, the PFS were both significantly improved in patients receiving T-Dxd and other novel ADCs compared with T-DM1. The most common grades 3-4 adverse events in the novel anti-HER-2 ADCs group were neutropenia (20.5%) and thrombocytopenia (28.1%) in the T-DM1 group.

**Conclusions:**

In patients with HER2-positive MBC previously treated with TKIs, both T-Dxd and other novel anti-HER2 ADCs yielded statistically significant better PFS than T-DM1 did, with tolerable toxicities.

## Teaser

There is little data showing which antibody-drug conjugates (ADCs) should be chosen for those patients whose treatment with tyrosine kinase inhibitors (TKIs) failed. This article analyzes the efficacy and safety between novel anti-human epidermal growth factor receptor 2 ADCs and trastuzumab emtansine for those with TKI treatment failure.

**Implications for Practice**

Antibody-drug conjugates (ADCs) have changed treatment patterns of human epidermal growth factor receptor 2 (HER2)-positive metastatic breast cancer. Previous studies such as DESTINY-Breast03 demonstrated clinical efficacy of trastuzumab deruxtecan for patients previously treated with trastuzumab. However, clinical trials paid less attention to the population prior treated with tyrosine kinase inhbitors (TKIs). This study focused on patients with TKI treatment failure, and the promising effect of novel anti-HER2 ADCs have been verified. These meaningful results suggest some clues for future studies in underrepresented populations of TKI treatment failure.

## Introduction

The emerging of antibody-drug conjugates (ADCs) has changed treatment patterns of human epidermal growth factor receptor 2 (HER2)-positive metastatic breast cancer (MBC).^1^ It is a new type of anti-tumor drug which combines monoclonal antibodies with cytotoxic drugs through linkers. ADCs used antibodies to target cancer cells and release cytotoxic drugs intracellularly to kill tumor cells with high efficiency and low toxicity.^2,3^ Mylotarg is the first-generation ADC drug approved for patients with acute myeloid leukemia (AML). As the second-generation ADCs, trastuzumab emtansine (T-DM1) was the anti-HER2 ADC for breast cancer. EMILIA study has confirmed the survival superiority of T-DM1 over lapatinib in the second-line treatment of HER-2 positive MBC.^4^ On this basis, T-DM1was the first HER2-targeting ADC approved by Food and Drug Administration (FDA) in 2013. Trastuzumab deruxtecan (T-Dxd) is the third generation of anti-HER2 ADCs drugs. The modified engineering structure further improved the function of ADCs.^5^ DESTINY-Breast03 study showed T-Dxd significantly prolonged the median progression-free survival (PFS) compared with T-DM1 for patients after progression on trastuzumab.^6^ Under this circumstance, T-Dxd has become a new standard treatment for HER2-positive breast cancer failed to trastuzumab.^7-9^

Despite the outstanding efficacy from ADCs, there is still a large population choosing tyrosine kinase inhibitors (TKIs) after trastuzumab due to the hard access to ADCs. Besides, the improvement of ADCs over lapatinib in HER2-positive metastatic breast cancer does not mitigate the superior efficacy of other target drugs like pyrotinib and neratinib.^10,11^ Previous real-world data also showed that pyrotinib achieved a longer survival than T-DM1 for patients with TKIs failure.^12^ Considering the cost-effectiveness, pyrotinib, instead of ADCs, is the level I recommendation for metastatic settings in Chinese Society of Clinical Oncology (CSCO) breast cancer guidelines even after approval of T-DM1 in 2021.^13^

There is no doubt to choose ADCs after TKIs. Unfortunately, clinical studies paid less attention to this kind of population. Nowadays, more than 700 ADCs are under research in the world, several promising ADCs are also under explorations. Choosing reasonable ADCs after TKIs has yet to be explored in HER2-positive MBC. Under these perquisites, we have conducted this real-world study to uncover the efficacy and safety of anti-HER2 ADCs after TKIs in HER2-positive breast cancer.

## Materials and Methods

### Study Population

This is a non-randomized, non-blinded real-world study in which all data are retrospectively collected by medical professionals from electronic medical record systems. Data collection was carried out from May 1, 2022 to June 30, 2022. Patients who met the following criteria in our department were enrolled in this study (Research number: CSCO BC RWS 2201). Patients were pathologically confirmed as invasive breast cancer with HER-2 (+++) or HER-2 (++) immunohistochemistry and FISH (+); there was at least 1 extracranial measurable lesion that met the definition of RECIST1.1 solid tumor efficacy evaluation criteria and at least one tumor evaluation after treatment; ADCs (including T-DM1, T-Dxd, and other ADCs under clinical studies) were used after TKIs (pyrotinib/lapatinib) failure; ECOG (Eastern Cooperative Oncology Group) score ≤ 2. All patients meeting those criteria should be included to avoid possible selection bias except for these who had mental abnormalities, combined with other tumors or severe comorbid conditions.

TKIs treatment failure was defined as discontinuation due to disease progression, unacceptable toxicity, or patient refused to treatment. Benefit from trastuzumab/TKIs was defined as gained complete response (CR) or partial response (PR) or stable disease (SD) lasting ≥ 6 months from trastuzumab/TKIs treatment.

This study was done in accordance with the principles of Good Clinical Practice, the Declaration of Helsinki, and Chinese regulations. The central ethics committee approved the study protocol.

### Treatment Protocol

All enrolled patients were divided into novel anti-HER-2 ADCs group and T-DM1 group according to the ADC regimen. Patients who both received T-DM1 and novel anti-HER2 ADC were regarded as novel anti-HER-2 ADCs group. T-Dxd (3.6 mg/kg q^3^w) was included in the novel anti-HER-2 ADCs group, as well as other anti-HER-2 ADCs in the pipeline such as MRG002 (2.6 mg/kg q^3^w),^14^ ARX788 (1.5 mg/kg q^3^w)^15,16^, and RC48 (2.0 mg/kg q^3^w).^17^ In the control group, T-DM1 was administered at 3.6 mg/kg q^3^w. Subsequent doses may be adjusted according to adverse drug reactions and patient’s tolerance.

### Efficacy and Safety Assessment

The primary endpoint for this study was PFS, which refers to the time from initiation of treatment to disease progression or death from any cause. The secondary study endpoints included objective response rate (ORR), clinical benefit rate (CBR), and safety. Efficacy was evaluated every 2 cycles according to Response Evaluation Criteria in Solid Tumors (RECIST, version 1.1). ORR was defined as the proportion of patients with CR and PR, and CBR was defined as the proportion of patients with CR, PR, and SD ≥ 6 months. Adverse reaction grading was evaluated according to the treatment records and laboratory test results with reference to common terminology criteria for adverse events (CTCAE) 4.0.

### Statistical Analysis

SPSS 19.0 was used for statistical analysis. Descriptive analysis was used to describe clinical characteristics. Continuous variables were compared by *t* test or Wilcoxon rank sum test, multiple group comparisons were performed by using an analysis of variance (ANOVA). χ^2^ test or Fisher exact probability method were used to compare categorical variables and differences in ORR and CBR between the 2 groups. Survival curves were plotted by using the Kaplan-Meier method and evaluated with the Log-rank test. COX regression analysis was applied to calculate the 95% confidence interval (CI) of hazard ratio (HR) values. All tests were 2 sided, and *P* < .05 was considered as statistically different.

## Results

### Clinical Characteristics

**Figure 1.**
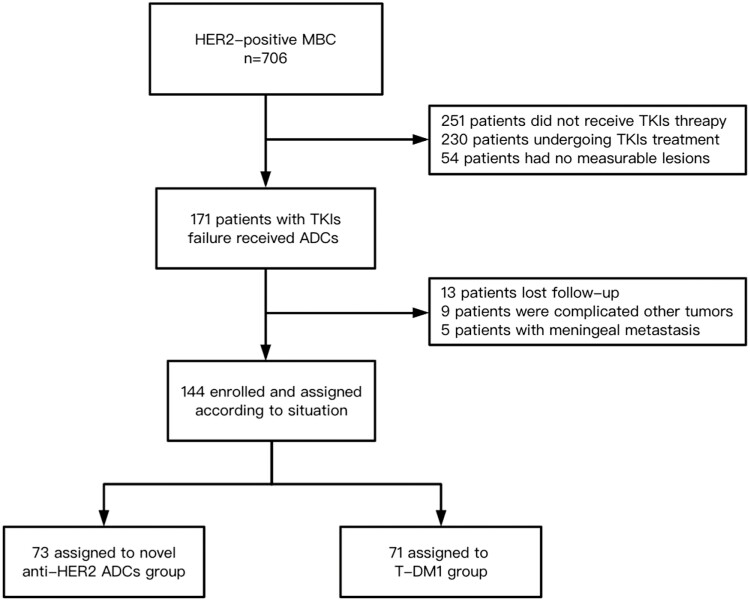
Study flow chart.

A total of 706 patients were diagnosed with HER2-positive metastatic breast cancer, 144 patients were included from 2013 to 2022 after excluding ineligible patients. The flow chart of study selection will be detailed in Fig. 1. The median age of the subjects was 45 years (range, 25-89 years), and all of them were female. There were 71 patients in the T-DM1 group and 73 patients in the novel anti-HER2 ADCs group, including 30 patients with T-Dxd, 24 patients with MRG002, 10 patients with ARX788, and 9 patients with RC48. Only 5 patients who used novel anti-HER2 ADCs after T-DM1 were grouped into novel ADCs group. Of the T-DM1 group, 71.8% (51/71) were aged < 50, a significantly higher frequency than that in novel anti-HER2 ADCs group (*P* = .017). The premenopausal patients in T-DM1 group account for 70.4% (50/71), this was significantly higher than that of novel anti-HER2 ADCs group (*P* = .006). Patient enrollment in different period were listed in Supplementary Fig. S1. 79.5% (58/73) and 16.9% (12/71) of patients were treated with trastuzumab + pertuzumab in novel anti-HER2 ADCs and T-DM1 group, respectively. All patients had previously received trastuzumab treatment, all patients in the novel anti-HER-2 ADCs group had previously received pyrotinib treatment, and 18 patients (24.6%) had received lapatinib treatment. In the T-DM1 group, 30 patients (42.3%) and 51 patients (71.8%) had received pyrotinib and lapatinib, respectively (Table 1).

**Table 1.** Characteristics of the patients at baseline (*n*, %).

Characteristic	Novel anti-HER2 ADCs (*n* = 73)	T-DM1 (*n* = 71)	*P*
Age (year)			.017
Median (range)	48 (25-89)	42 (25-63)	
<50	38 (52.1)	51 (71.8)	
≥50	35 (47.9)	20 (28.2)	
Hormone receptor status			.73
Positive	38 (52.1)	39 (54.9)	
Negative	35 (47.9)	32 (45.1)	
Menopausal status			.006
Premenopausal	35 (47.9)	50 (70.4)	
Postmenopausal	38 (52.1)	21 (29.6)	
Clinical stage at diagnosis			.941
Ⅰ-Ⅲ	61 (83.6)	59 (83.1)	
Ⅳ	12 (16.4)	12 (16.9)	
Visceral metastases			.96
Yes	67 (91.8)	65 (91.5)	
No	6 (8.2)	6 (8.5)	
Metastatic site			
Bone	41 (56.2)	40 (56.3)	.983
Liver	40 (54.8)	37 (52.1)	.747
Lung	42 (57.5)	35 (49.2)	.322
Brain	20 (27.4)	19 (26.8)	.932
Number of metastatic sites			.658
1	8 (11.0)	6 (8.5)	
2	18 (24.6)	22 (31.0)	
≥3	47 (64.4)	43 (60.5)	
Number of previous lines of anti-HER2 therapy			.760
≤3	28 (38.4)	30 (40.3)	
>3	45 (61.6)	41 (57.7)	
Previous anti-HER2 therapy			
Trastuzumab	73 (100.0)	71 (100.0)	—
Trastuzumab + pertuzumab	58 (79.5)	12 (16.9)	<.001
Pyrotinib	73 (100.0)	30 (42.3)	<.001
Lapatinib	18 (24.6)	51 (71.8)	<.001

Abbreviations: HER2: human epidermal growth factor receptor 2; ADCs: antibody-drug conjugates; T-DM1: trastuzumab emtansine.

### Efficacy

**Figure 2.**
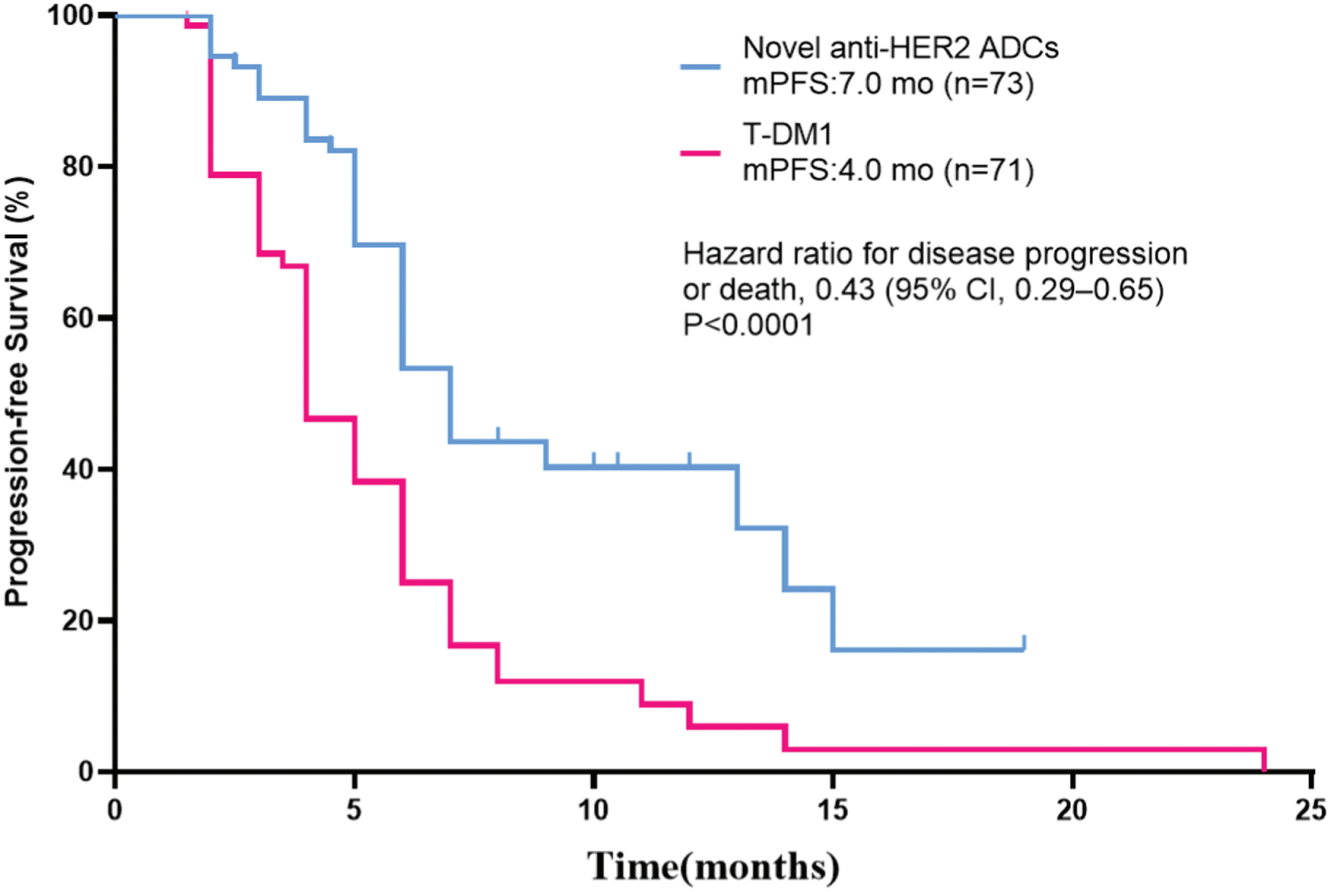
Kaplan-Meier analysis of progression-free survival (PFS) for all patients treated with novel anti-HER2 ADCs and T-DM1. HER2: human epidermal growth factor receptor 2; ADCs: antibody-drug conjugates; T-DM1: trastuzumab emtansine; mo: month.

**Figure 3.**
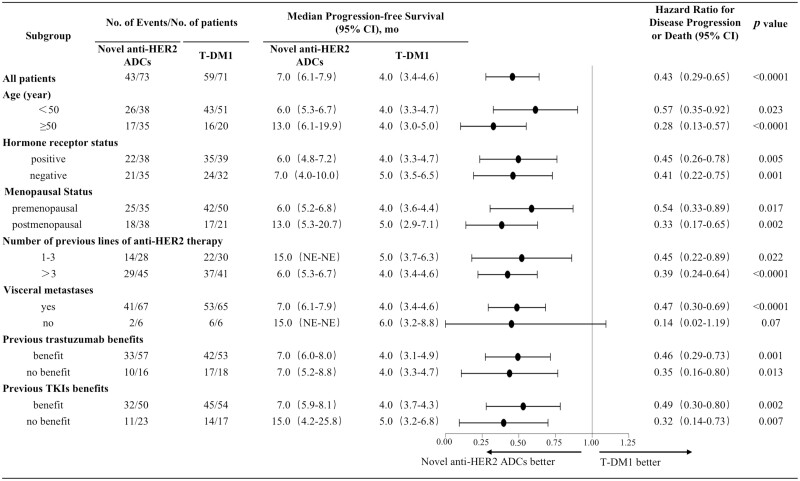
Subgroup analysis of progression-free survival (PFS). HER2: human epidermal growth factor receptor 2; ADCs: antibody-drug conjugates; T-DM1: trastuzumab emtansine; mo: month; TKIs: tyrosine kinase inhibitors; 95% CI: 95% confidence interval.

At the time of the last follow-up, 43 patients (58.9%) in the novel anti-HER2 ADCs group and 59 patients (83.1%) in the T-DM1 group reached the endpoint. The median PFS of the 2 groups were 7.0 months (95% CI, 6.1-7.9 months) and 4.0 months (95% CI, 3.4-4.6 months), *P* < .0001 (Fig. 2). Compared with the T-DM1 group, the study group reduced the risk of disease progression or death by 57%, HR = 0. 43 (95% CI, 0.29-0.65), *P* < .0001. Age, hormone receptor status, menopausal status, number of previous anti-HER2 treatment lines, visceral or non-visceral metastases, benefit from previous trastuzumab treatment or not, and benefit from previous TKIs treatment or not were included in the subgroup analysis. The results showed that except for no visceral metastasis, the benefits of PFS in other subgroups were consistent with those of the overall population, such as previous lines of anti-HER2 lines ≤ 3 (15.0 months vs. 5.0 months; stratified HR = 0.45, 95% CI, 0.22-0.89; *P* = .022); visceral metastases (7.0 months vs. 4.0 months; stratified HR = 0.47, 95% CI, 0.30-0.69; *P* < .0001) (Fig. 3).

As shown in Table 2, no patients in either group achieved CR. The PR rates of anti-HER2 ADCs group and T-DM1 group were 54.8% and 22.5%, and the SD rates were 39.7% and 69.0% respectively. ORR and CBR were significantly higher in the study group compared with the control group. The ORR was 54.8% versus 22.5% (*P* < .0001), and the CBR was 65.8% vs. 47.9% (*P* = .03).

**Table 2.** Comparison of efficacy between novel anti-HER2 ADCs and T-DM1.

Type of response no. (%)	Novel anti-HER2 ADCs (*n* = 73)	T-DM1 (*n* = 71)	*P*
CR	0	0	
PR	40 (54.8)	16 (22.5)	
SD	29 (39.7)	49 (69.0)	
SD ≥ 6	8 (11.0)	18 (25.4)	
PD	4 (5.5)	6 (8.5)	
ORR	40 (54.8)	16 (22.5)	<.001
CBR	48 (65.8)	34 (47.9)	.03

Abbreviations: HER2: human epidermal growth factor receptor 2; ADCs: antibody-drug conjugates; T-DM1: trastuzumab emtansine; CR: complete response; PR: partial response; SD: stable disease; PD: progressive disease; ORR: objective response rate; CBR: clinical benefit rate.

**Figure 4.**
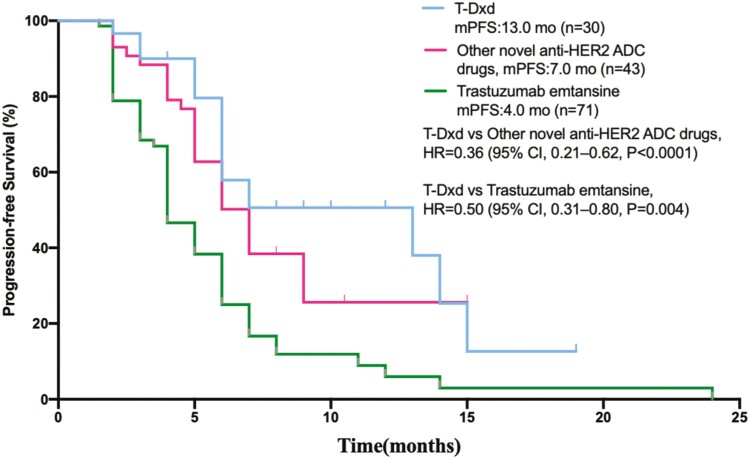
Kaplan-Meier analysis of progression-free survival (PFS) for all patients treated with T-Dxd, other novel anti-HER2 ADCs and T-DM1. T-Dxd: trastuzumab deruxtecan; HER2: human epidermal growth factor receptor 2 (HER2); ADCs: antibody-drug conjugates; T-DM1: trastuzumab emtansine; mo: month.

Patients were further divided into 3 groups in the subgroup analysis, including 30 cases in the T-Dxd group, 43 cases in the other novel anti-HER2 ADCs group and 71 cases in the T-DM1 group. The efficacy of the T-Dxd group was significantly better than the other 2 groups, and the median PFS in the T-Dxd group, other novel anti-HER ADCs group, and T-DM1 group was13.0 months (95% CI, 8.1-12.5 months), 7.0 months (95% CI, 6.2-9.8 months), and 4.0 months (95% CI, 4.4-6.8 months), respectively. There were statistical differences among the 3 groups (*P* < .0001) (Fig. 4).

### Safety

The incidence of adverse events in the novel anti-HER2 ADCs group and the T-DM1 group is shown in Table 3. The overall incidence of adverse events was similar between the 2 groups. The incidence of gastrointestinal disorders and fatigue was higher in the novel anti-HER2 ADCs group, nausea (76.7%), vomiting (46.5%), and fatigue (65.8%); the main adverse reactions in the T-DM1 group were thrombocytopenia (53.5%) and fatigue (49.3%). Grades 3-4 adverse reactions in the novel anti-HER2 ADCs group were mainly neutropenia (20.5%), nausea (12.3%), and vomiting (11.0%), while grades 3-4 adverse reactions in the T-DM1 group were mainly thrombocytopenia (28.1%). Interstitial lung disease (ILD) occurred in both groups, and the incidence of the novel anti-HER2 ADCs group was higher than that of the T-DM1 group (5.4% vs. 1.4%).

**Table 3.** Treatment-related adverse events in the 2 groups (*n*, %).

Adverse event	Novel anti-HER2 ADCs (*n* = 73)		T-DM1 (*n* = 71)	
	Any grade	Grade3~4	Any grade	3~4
Neutropenia	33 (45.2)	15 (20.5)	13 (18.3)	4 (5.6)
Leukopenia	26 (35.6)	6 (8.2)	13 (18.3)	2 (2.8)
Anemia	28 (38.4)	5 (6.8)	15 (21.1)	4 (5.6)
Thrombocytopenia	11 (15.1)	3 (4.1)	38 (53.5)	20 (28.1)
Diarrhea	13 (17.8)	6 (8.2)	6 (8.5)	0
Constipation	11 (15.1)	0	5 (7.0)	0
Nausea	56 (76.7)	9 (12.3)	5 (7.0)	0
Vomiting	34 (46.5)	8 (11.0)	3 (4.2)	0
Fatigue	48 (65.8)	4 (5.4)	36 (49.3)	2 (2.8)
Decreased appetite	31 (42.4)	0	14 (19.7)	0
Elevated transaminase	11 (15.1)	2 (2.7)	16 (22.5)	4 (5.6)
Peripheral Neuropathy	20 (27.4)	3 (4.1)	6 (8.5)	0
Interstitial lung disease	4 (5.4)	1 (1.4)	1 (1.4)	0

Abbreviations: HER2:, human epidermal growth factor receptor 2; ADCs: antibody-drug conjugates; T-DM1: trastuzumab emtansine.

## Discussion

This real-world study compared the efficacy and safety between novel anti-HER2 ADCs and T-DM1 in patients with TKI treatment failure, and the results of this study contributed data to the TKIs failure population. To our knowledge, this is the first real-world study to systematically analyze the efficacy and safety of different ADCs after TKIs. Our results have several troubling implications. First, patients in novel anti-HER2 ADCs group had a longer PFS than that in T-DM1 group, and improvement were also observed in ORR and CBR. Subgroup analyses identified all subgroups were consistent with those of the overall study population. Second, for these patients using novel anti-HER2 ADCs, T-Dxd showed a superior survival than other ADCs, while other novel ADCs also achieved a superior survival benefit than T-DM1 did. In addition, our study confirmed a statistics significance of AEs in these novel ADCs when compared with that in T-DM1.

From this study, we found novel anti-HER2 ADCs significantly improved the PFS than T-DM1 in patients after TKI treatment failure. This improvement also made sense in ORR and CBR. Numerous articles have reported that patients treated with T-DM1 after trastuzumab and lapatinib had approximately 4.6 to 6.9 months of median PFS, providing valuable insights for us. T-DM1 in this study achieved a similar PFS compared with the above results.^18-20^ However, in novel ADCs group, the survival is much lower than other randomized studies reported. The survival difference describes the substantial influence from the real-world.^21^ Two reasons may be responsible for this disparity. For one thing, more than 90% of patients had visceral metastases compared with 70% in DESTINY-Breast03 study. The increased tumor burden could have a negative influence on PFS as well as CBR and ORR.^22^ For another, there were only less than half of the patients using T-Dxd in novel anti-HER2 ADCs group. The efficacy of other novel anti-HER2 ADCs drugs under investigation is not clear, which would underestimate the actual efficacy of these novel ADCs on cancer patients. To be noted, the proportion of premenopausal patients was uneven. Although there is no evidence to suggest that menopausal status would affect the choice of anti-HER2 ADC, the longer enrollment time of T-DM1, compared with T-Dxd, would have a positive impact on ADC selection. Further studies should be carried out.

To further analyze the efficacy of different novel anti-HER2 ADCs, they were divided into T-Dxd and other novel ADCs. The survival of T-Dxd was significantly longer than other novel ADCs and T-DM1. Meanwhile, patients received other novel ADCs also achieved a longer PFS than T-DM1 did. From this respective, T-Dxd may be the first choice of patients after TKI treatment failure, other novel ADCs are rational options than T-DM1 when T-Dxd is not available.

Subgroup analysis further confirmed the survival benefits across key subgroups. The benefit was also evident in some higher risk features, such as visceral metastases. The insufficient sample size, especially in these patients without visceral metastasis subgroup, resulted in lower statistical power and negative result. Regardless of benefit from previous TKIs, PFS improvement was seen in novel ADCs group. Compared with T-DM1, the optimization of antibody engineering technology enhanced anticancer activity and bystander effect of these novel ADCs.^23,24^ Besides, the treatment of TKIs was not well balanced in these population. All patients were treated with pyrotinib in novel ADCs group, while only 42.3% treated in T-DM1 group. These differences further confirmed the advantages of novel ADCs for patients prior treated with pyrotinib. However, no interactive analysis was performed to further demonstrate the statistical significance of the group differences, and the finding should be interpreted with caution.

Safety management might be another restriction for promotion of those ADCs. As we have shown in this study, gastrointestinal and hematological adverse reactions were common adverse events in both groups. There were a few noteworthy differences in grades 3-4 adverse reactions, the hematological toxicity of novel ADCs was mainly neutropenia, while T-DM1 was thrombocytopenia. These differences were mostly related to the payload of different ADCs.^2,23^ For example, the novel ADCs group mainly presented with gastrointestinal toxicity and myelosuppression. This was consistent with the toxicity profile of camptothecin and auristatin, which were payloads of novel ADCs. While the payload of T-DM1 was maytansinoid who has a more pronounced effect on thrombocytopenia and elevated transaminase. ILD is another severe adverse event caused by ADCs. In some pooled analysis, the incidence of ILD could be as high as 16.8%.^25,26^ Indeed, the incidence of ILD in novel ADCs was 5.4%. The decrease in incidence can be explained by the application of management guidelines. Nevertheless, it is noteworthy that higher incidence of ILD in novel ADCs compared with T-DM1.

As the real-world study to investigate the efficacy of ADCs on for HER2-positive patients after TKI treatment failure, there were also some limitations, such as a small sample size, inevitable selection bias, and underestimated adverse events. Although this retrospective observational study was based on a single center database, it is not weakened by lack of randomization between 2 groups attributed to the comparability in our pairwise comparison within individuals. Another multicenter real world study has been conducted to verify the real efficacy of novel ADCs after TKI. Despite these limitations, the results of this study are still crucial to provide reference for the optional treatment strategy of HER2-positive MBC after TKI treatment failure.

## Conclusion

In patients with HER2-positive MBC previously treated with TKIs, both T-Dxd and other novel anti-HER2 ADCs yielded statistically significant better PFS than T-DM1 did, with tolerable toxicities. The novel ADCs are preferred option for HER2-positive refractory MBC after TKI treatment failure.

## Supplementary Material

oyad127_suppl_Supplementary_Figure

## Data Availability

The data underlying this article will be shared on reasonable request to the corresponding author.
