# Supplementary figures and images for: Novel Anti-HER2 Antibody-Drug Conjugates Versus T-DM1 for HER2-Positive Metastatic Breast Cancer After Tyrosine Kinase Inhibitors Treatment
Source: Oncologist. 2023 May 22;28(10):e859–66. doi: 10.1093/oncolo/oyad127 (PMC10546815; doi:10.1093/oncolo/oyad127)

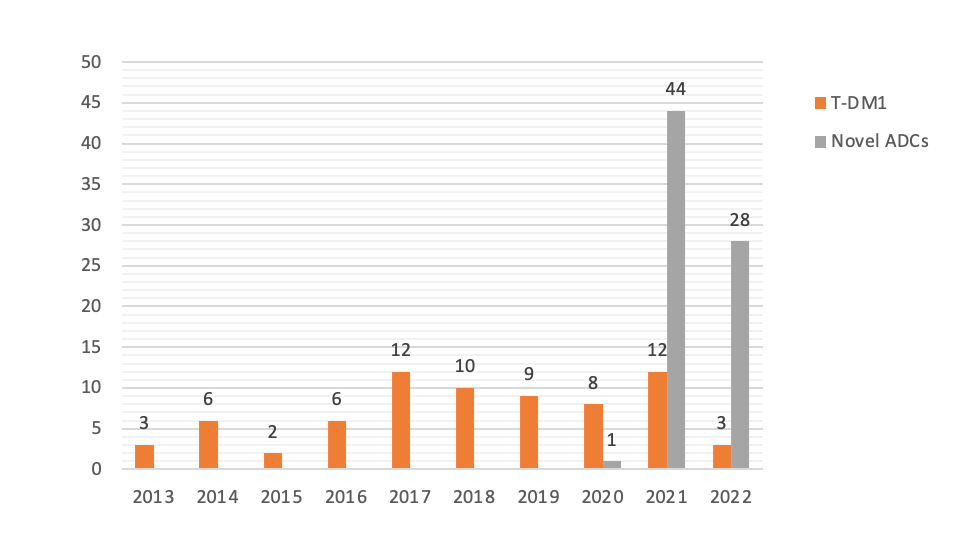


supplemental figure. patients enrollment time

Supplement: oyad127_suppl_Supplementary_Figure [file oyad127_suppl_supplementary_figure.docx]
